# Supplementary material for: Electronic health record analysis identifies kidney disease as the leading risk factor for hospitalization in confirmed COVID-19 patients
Source: PLoS One. 2020 Nov 12;15(11):e0242182. doi: 10.1371/journal.pone.0242182 (PMC7660530; doi:10.1371/journal.pone.0242182)
Supplement: S1 Methods — (PDF) [file pone.0242182.s002.pdf]

S1 Methods.

Definition of EHR derived clinical traits used in Table 1.

- BMI
  - Evaluated after the age of 18 and not during pregnancy spans.
  - Heights are the median value of all heights for the individual after the age of 18.
  - Each weight is then evaluated with any weight < 50 or > 700 being thrown out.
  - Mean lifetime (EHR) BMI is used

- Smoking Status
  - History = Record of smoking status in EHR
  - Current = Most recent smoking status in EHR

- Chronic Kidney Disease
  - Active CKD diagnosis on patient's problem list or most recent eGFR < 15
    - Stratified by Stage
  - ESRD diagnosis or eGFR criteria considered Stage V

- Chronic Lung Disease

  Active CLD diagnosis on patient's problem list

- Diabetes
  - Two or more of the following three criteria are satisfied:
    - Active diabetes diagnosis on patient's problem list
    - Active antidiabetic outpatient medication order or medication reconciliation entry
    - ≥50% of outpatient labs abnormal in last 2 years
      - Fasting glucose ≥126
      - Non-fasting glucose ≥ 200
      - HbA1c ≥ 6.5

- Heart Failure
  - Active heart failure diagnosis on patient's problem list
  - Heart failure diagnosis as at least 1 encounter with any HF ICD-9 or ICD-10 codes and prescription of HF medication

- Hypertension
  - Two or more of the following three criteria are satisfied:
    - Active HT diagnosis on patient's problem list
    - Active antihypertensive outpatient medication order or med reconciliation entry
    - ≥50% of outpatient BP's measured in last two years are elevated
      - Diastolic BP ≥ 90 or Systolic BP ≥ 140
- Pneumonia
  - Active problem list diagnosis entry of Pneumonia

- Respiratory Distress
  - Acute Respiratory Distress Syndrome (ARDS) or Respiratory Distress noted on the problem list diagnosis in EHR
