# Supplementary material for: Electronic health record analysis identifies kidney disease as the leading risk factor for hospitalization in confirmed COVID-19 patients
Source: PLoS One. 2020 Nov 12;15(11):e0242182. doi: 10.1371/journal.pone.0242182 (PMC7660530; doi:10.1371/journal.pone.0242182)

S1 Fig: Study flow diagram**.**

SARS-CoV-2=severe acute respiratory syndrome coronavirus 2; COVID-19 severe acute respiratory syndrome coronavirus 2.

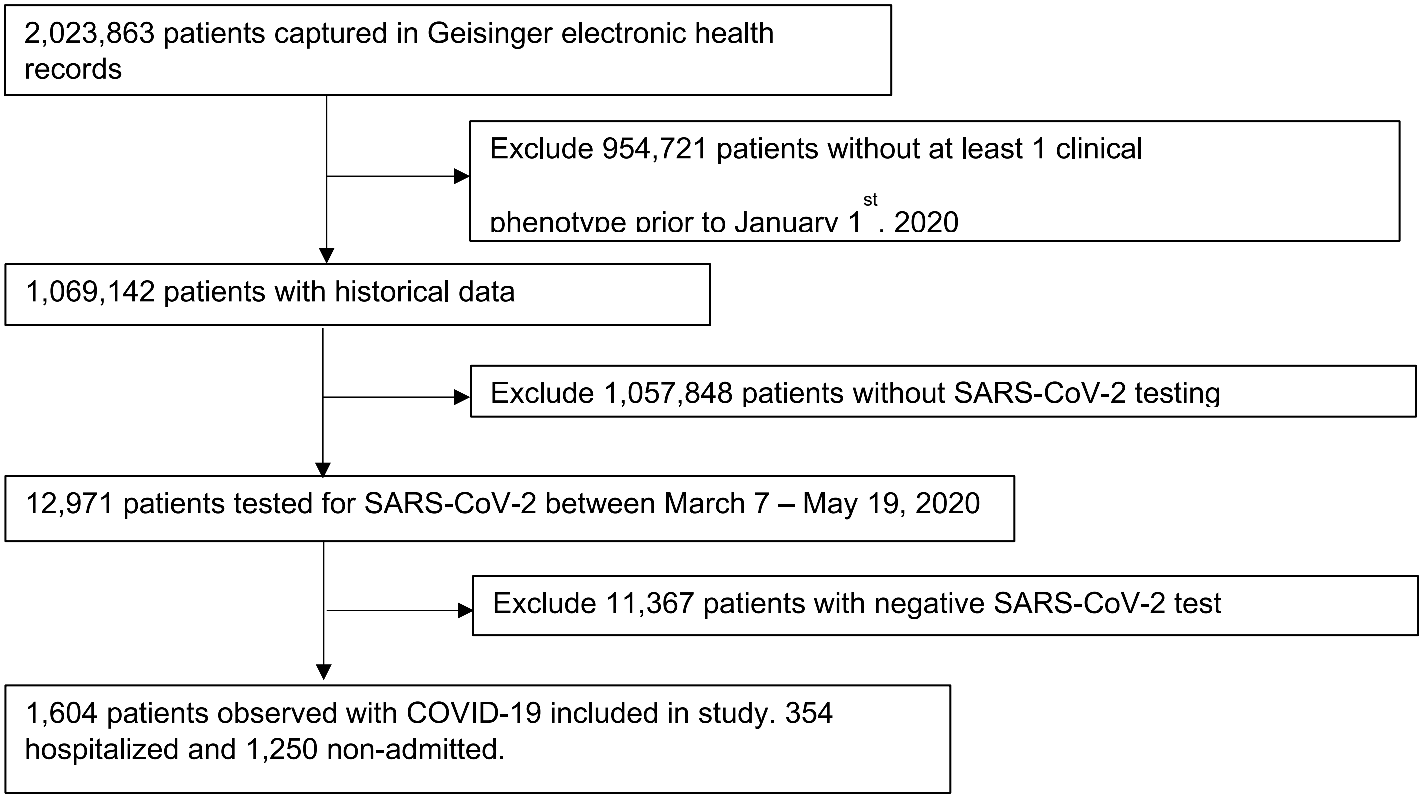

Supplement: S1 Fig — (PDF) [file pone.0242182.s003.pdf]
