# Supplementary material for: Electronic health record analysis identifies kidney disease as the leading risk factor for hospitalization in confirmed COVID-19 patients
Source: PLoS One. 2020 Nov 12;15(11):e0242182. doi: 10.1371/journal.pone.0242182 (PMC7660530; doi:10.1371/journal.pone.0242182)

S2 Fig:

Prevalence of validated disease phenotypes using EHR data among the total EHR population, all those tested for COVID-19, those who tested negative for COVID-19, COVID-19(+) individuals not needing admission and hospitalized for COVID-19(+) individuals. Numbers above bars are percentage for each group.

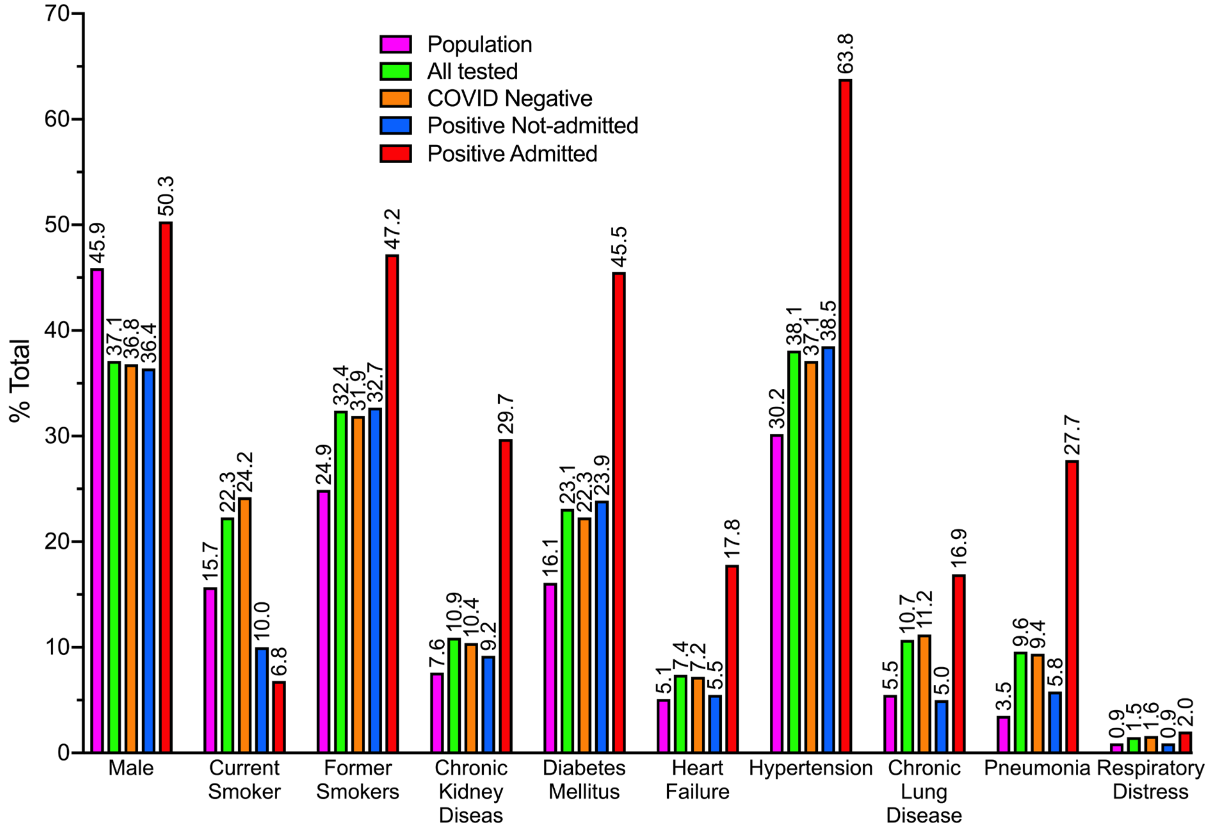

Supplement: S2 Fig — (PDF) [file pone.0242182.s004.pdf]
