# Supplementary material for: Electronic health record analysis identifies kidney disease as the leading risk factor for hospitalization in confirmed COVID-19 patients
Source: PLoS One. 2020 Nov 12;15(11):e0242182. doi: 10.1371/journal.pone.0242182 (PMC7660530; doi:10.1371/journal.pone.0242182)
Supplement: Attachment — Submitted filename: Response_to_REVIEW_COMMENTS.docx [file pone.0242182.s005.docx]

PONE-D-20-26257
Electronic health record analysis identifies kidney disease as the leading risk factor for hospitalization in confirmed COVID-19 patients.
PLOS ONE

We would like to thank the two reviewers for the insightful, and positive review of our manuscript. We have addressed all the points raised and edited the manuscript to reflect the changes as suggested by reviewers. Point by point response to each of the questions is denoted in red below.

Sincerely,

Tooraj Mirshahi

Reviewer #1: Authors pursue a very interesting and commendable endeavor of PheWAS of CoVID-19 positive patients to identify those at high risk for CoVID-related hospitalization. Following are our comments associated with each section:

Introduction

Line 6 regarding ‘Case reports have shown that older age, hypertension and diabetes are risk factors for COVID-19-related complications’. Would suggest omitting this statement (case reports) since there are a plethora of studies now available that demonstrate risk factors associated with CoVID-19 related complications. Would suggesting at least 2-3 major studies. For example, the study you quoted as reference [10].

We have updated this sentence in the introduction and included additional citations.

Results

1. Second paragraph- would replace ‘acute renal failure’, with ‘acute kidney injury’ as terminology. Would be prudent to separate out pre-existing and new acute condition, since the intent is to find risk factors for CoVID related hospitalizations.

Thank you for the suggestion on terminology, which has been updated. As our desire was to determine what pre-existing conditions were most strongly associated with increased risk of COVID-19 hospitalization, we focused on pre-existing conditions prior to 1/1/2020, and thus did not look at new acute conditions such as AKI occurring at the same time of COVID-19-related hospitalization for this study.

2. Also is the AKI at the time of diagnosis of CoVID-19 and how have you defined an AKI? The Methods specify ‘last outpatient serum creatinine value’, which could be very variable since most patients may not have had a creatinine checked proximal to diagnosis of CoVID-19. As per most recent data, AKIs in context of CoVID-19 have only developed during hospitalization course, rather than at the time of diagnosis. Please clarify your definition of AKI.

Potential risk factor phenotypes, including AKI, were defined using PheCodes mapped to ICD codes as described in the methods (<https://phewascatalog.org/phecodes_icd10cm>) prior to 1/1/2020. We did not examine AKI outcomes at the time of hospitalization in this study or use creatinine-based AKI definitions, but agree this is a highly important question. Future analyses are planned to rigorously examine risk factors of AKI among patients with COVID-19 and long-term outcomes.

3. Second paragraph- how did you define ESKD as well, since you have mentioned CKD stage 5 and ESKD together. Please clarify.

As mentioned previously, we used the PheCodes Map 1.2, which had previously organized these codes together (<https://phewascatalog.org/phecodes_icd10cm>). This includes ICD-10 code N18.5 (Chronic kidney disease, stage 5) and N18.6 (End stage renal disease). Realizing that ICD codes have limitations, we did additional sensitivity analyses as described in the paper to verify patients were on dialysis or had kidney transplant.

4. Second paragraph- please explain what encompasses the term ‘nonhypertensive congestive heart failure’. A slightly non-descript term and might be confusing to the reader.

Thank you for this suggestion. We agree this terminology is confusing and came from PheCodes Map 1.2. These PheCodes sometimes have overlap, and sometimes are very specific. We have reiterated this fact in the paper hoping to reduce the confusion, while keeping the ICD code standard used in PheCode Map 1.2.

5. Second paragraph- you have listed pneumonia as a risk factor. Again, an acute event and is this also present at the time of diagnosis of CoVID-19.

As mentioned before, these were based on diagnoses prior to 1/1/2020 rather than acute events.

6. On page 4 of the manuscript, there is a typo for number 15 following eGFR 15-29: 3) eGFR 15-29 15 ml/min/1.73m2 without kidney transplant;

This has been corrected.

Discussion

1. First paragraph- ‘However, initial reports of COVID-19 describing potential risk factors for hospitalization and other adverse outcomes have often been derived from cohorts without sufficient pre-hospitalization data that is often incomplete or missing’. Unfortunately, this is now an inaccurate statement- you could say that in March or April 2020. But as I quoted an example in the introduction section, there a good few studies now available looking at hospitalization risk and adverse outcome. Here’s another large study of >300,000 in PNAS (<https://www.pnas.org/content/early/2020/08/10/2011086117>). More- <https://academic.oup.com/cid/advance-article/doi/10.1093/cid/ciaa1012/5872581>,
<https://www.ncbi.nlm.nih.gov/pmc/articles/PMC7245300/>

Please consider updating the manuscript. Your study certainly does add to the literature of risk factors, and you could elucidate that in the discussion section.

Thank you for this comment. We agree COVID-19 literature has changes, so we removed sentence under question and added these citations to the text elsewhere.

2. First paragraph- ‘Further, confounders such as age, sex, and race are often not accounted for’. Again, would be inaccurate to state that as well. Please consider omitting, based on examples discussed above. Another example- <https://bmcinfectdis.biomedcentral.com/articles/10.1186/s12879-020-05144-x>
<https://www.nejm.org/doi/full/10.1056/NEJMsa2011686>

We have removed the text about confounders in the discussion as the reveiwer correctly mentions some of the newer studies consider these confounders we have added some of these studies as new citations.

3. First paragraph- ‘As well as the CMS reports showing higher risk of hospitalization among ESKD patients (not adjusted for covariates). Our finding of high risk of hospitalization in kidney transplant patients mirrors that of a case series of 36 consecutive kidney transplant patients at Montefiore where 28/36 (78%) were hospitalized’,

Again, multiple studies have since been been published regarding CoVID-19 in ESRD/ESKD patients and kidney transplant recipients.

Would consider discussing studies such as <https://www.kidney-international.org/article/S0085-2538(20)30945-5/fulltext>, for ESKD in addition to CMS reports.

Here are bigger studies on kidney transplant recipients for the discussion. The Montefiore study is small and is from early days of COVID-19- <https://www.kidney-international.org/article/S0085-2538(20)30961-3/fulltext#.X0OiqBDfdDg.twitter>

<https://onlinelibrary.wiley.com/doi/full/10.1111/ajt.16185>

We have added these citations to our study.

Again, consider updating the manuscript with these more recent studies.

4. First paragraph- ‘The findings reported here identify pathophysiologies that impact the clinical course of COVID-19 and may be used to identify individuals at greatest risk for COVID-19-related complications’. The term ‘pathophysiologies’ seems misleading – consider using ‘co-morbidities’ instead.

Pathophysiologies has been replaced with co-morbidities.

5. Second paragraph- ‘How clinical conditions increase the risk of COVID-19-related complications is not known’. Again, as discussed before. Not necessarily an accurate statement as knowledge has quickly evolved. Consider updating or omitting.

We agree that much more is now known about clinical condition that increase risk for COVID-19 related complication; however, there are many remaining questions, so we updated the sentence to reflect both these this facts.

6. Third paragraph- ‘Our findings also have implications for studies that seek to find genetic variants that alter the course of the disease (e.g. <https://www.covid19hg.org/>). We suspect that some genetic variants will be associated with these traits based on their effects on pre-existing clinical conditions’. We are not certain how this statement regarding genetic studies fits in the discussion. Consider clarifying or omitting.

We omitted this phrase.

7. We tend to agree with the authors that one of the most important limitations of their study is generalization as their population is mostly Caucasian. Also, consider adding race to table 1 of participants characteristics.

We added a citation and sentence in the discussion about this point.

We hope these few comments will help the authors improve on their interesting manuscript and we thank the editor for entrusting our opinion.

Reviewer #2: This is an interesting study adding important information on the importance of chronic kidney disease as risk factor for COVID-19 associated hospitalization rate in the USA.

Comments:
Of high interest would be the outcome of the 354 patients admitted to the hospital. It would be very interesting to provide data on the outcome of those patients after hospitalisation, divided according to the different diseases/phenotypes (outcome: admission to ICU, need for ventilation, fatal outcome)? Did CKD patients (e.g. dialysis patients or stage IV and V CKD patients) have a higher risk for disease progression or death?

A table of available outcomes has been added (Now Table 3) to compare CKD vs. Non-CKD patients. This data is from all those who were admitted to the hospital, regardless of whether they were indcluded in the PheWAS (since PheWAS had a more stringent indclusion criteria based on presence of longer EHR data). Death rate is higher among those with known history of CKD (data is in the table and in the results). However, CKD patietns are older and have higher burden of co-morbid conditions. We cannot further subdivide the CKD among this group as we do not have comprehensive data on all of them. Table 4 has specific data on patients with confirmed stages of CKD.

The high OR of 11 for hospitalisation with COVID-19 for patients with stage 5 CKD and/or ESKD is astonishing, especially when compared to other phenotypes, such as CHF. This point was not really addressed in the discussion. The renal tropism of SARS-CoV-2 and the high ACE2R expression in the kidneys are probably not part of the explanation. It would be interesting to have some discussion on this.

As seen in Table 3, patients with CKD have many comorbidities, which likely contribute to their increased risk of hospitalization. These comorbid conditions are perhaps even higher among the ESKD patients. The increased risk could also be related to greater susceptibility to infection due to decreased immune function.

Additionally, what triggered the hospitalisation for those patients with COVID-19? Could it be that patients with ESKD were more likely to be hospitalized for fear of progression to a more serious disease and perhaps because of a strong interest in isolating these patients from other dialysis patients and keeping them away from dialysis units rather than because of the severity of COVID-19 disease? Since hospitalization was the endpoint of this study, there should be some comments on the reasons/triggers for hospitalization.

Hospitalization was based on presence of symptoms, as described by the CDC at the time of admission. The enrichment of hospitalized ESKD patients is not due to fear of progression as our nephrologists were extremely concerned about nosocomial infection and testing was done very selectively for patients with symptoms, so there was no increased any desire to isolate patients. In addition, hospitals in our system strived to discharge patients expeditiously even with COVID-19. We established policies throughout our dialysis units in the region to ensure patients could be discharged quickly out of the office to designated COVID-19 shifts in our dialysis units.

Table 1:
As reported in the text, there were 1,604 persons tested positive for SARS-CoV-2. But in table 1 only 1,250 positive patients are reported. Please correct.

We apologize about the confusion. The 1250 is those who were positive but not admitted, 354 were admitted. (1250+354=1604).

Please explain the meaning of the numbers in the first line (inclusion Criteria, N (%)). Percent of what? Of 18,372 persons with testing for SARS-CoV-2 positivity, 12,971 fullfilled inclusion criteria of this study. This is 70.6% of all tested persons. The number of persons with fullfilling inclusion criteria and a negative SARS-CoV-2 Test were 11,367. But what does 72,4% refer to? It should be either 61,9% of the tested population or 87,6% of the tested population with inclusion criteria. Please, re-check your calculations and numbers of this table, especially line 1 and please explain.

We apologize of the confusion caused here and have updated this table and added new information in the results to clarify soma of the confusion. In summary, we had 18,372 tested individuals, 15707 were negative, 2665 were positive, and 565 were admitted. Not all of these patients met the inclusion criteria for PheWAS, so the number in the table reflect the patients included in PheWAS (these are described in the methods and not briefly in the results).

Table 2:
Almost 50% of all CKD cases are „unspecified“ regarding to CKD stage (line 1). Since CKD stage seems to have tremendous impact on the COVID-19 ass. hospitalization risk, it would be most welcome if those 172 „unspecified stage “ patients could be re-classified according to eGFR data and added to each staged CKD phenotypes? In table 3 it is said, that eGFR data are available for 1393 individuals…

As mentioned above, these PheCodes are imperfect for CKD. Optimally, CKD is staged using the eGFR data, which was done for Table 3. Since the value of doing a PheWAS is to do an unbiased analysis of phenotypes associated with COVID-19 hospitalization, we used the PheCodes as published. We have included the ICD-codes associated with the PheCodes to make it easier for readers to understand what ICD-codes comprised each PheCode.

The small number of patients with stage IV and V/VD CKD lowers the power of this analysis. It does not seem biologically plausible that the Odds for hospitalisation with COVID-19 increases from 2,68 with CKD III to 11,85 with CKD IV.
In table 3 the numbers are much more plausible with the association of COVID-19 related hospitalization with eGFR stages. Stage III had an OR of (adj) 1,03, stage IV 2,90 and stage V 8,83. In table 3 unadjusted and adjusted OR were given. Please confirm that ORs in table 2 are adjusted values (should be also stated in the table legend)

We agree the limited number of patients is a limitation. However, we respectfully disagree with the comment about biological plausibility since patients with stage 4 CKD are considerably sicker than those with stage 3 CKD. It may be that the associated comorbidities contribute significantly. This has been added to the discussion. These are adjusted age, sex, race/ethnicity. This has been added to the table legend.

Figure 1:
please explain why there are three (!) circles for chronic kindey diseases at different risk points in the graph (and two circels for acute renal faliure as well). This is confusing and should be clarified.

As mentioned above, PheWAS uses PheCode that are mapped to specific ICD codes. These PheCodes sometimes have overlap, and sometimes are very specific. We have reiterated this fact in the paper and in the legend hoping to reduce the confusion. We have referenced the source of PheCode where one can look up specific ICD codes associated with each one. the PheCode in the figure

Further comments:
In the discussion the two terms "ESRD" and "ESKD" are used. This should be harmonized.
(literature suggestion on nomenclature in kidney diseases: NDT, Vol 35, 2020, 1077–1084, https://doi.org/10.1093/ndt/gfaa153

Thank you this has been updated.

I suggest not to use the term „COVID-19 positive patients“, neither in the txt nor in table one. It should be either „SARS-CoV-2 positive“ or „SARS-CoV-2 infected patients“ or „patients with COVID-19“. ´COVID-19 is not a virus (SARS-CoV-2 is…), it’s a disease.

Thank you, we have replaced COVID-19 with SARS-CoV-2 where appropriate.
